# BiTEs, DARTS, BiKEs and TriKEs—Are Antibody Based Therapies Changing the Future Treatment of AML?

**DOI:** 10.3390/life11060465

**Published:** 2021-05-23

**Authors:** Cecily Allen, Amer M. Zeidan, Jan Philipp Bewersdorf

**Affiliations:** Section of Hematology, Department of Internal Medicine, Yale University School of Medicine, New Haven, CT 06520-8028, USA; cecily.allen@yale.edu (C.A.); amer.zeidan@yale.edu (A.M.Z.)

**Keywords:** acute myeloid leukemia, AML, bispecific antibody, BiTE, DART, safety

## Abstract

Nearly four decades after their conceptualization, antibody-based therapies are slowly being added to the treatment landscape of acute myeloid leukemia (AML). While the antibody–drug conjugate gemtuzumab ozogamicin is the only antibody-based therapy that has been approved for AML treatment thus far, several bispecific antibodies have been developed and shown early encouraging results. Bispecific antibodies comprise a wide variety of constructs that share the common concept of simultaneous binding of a surface target on malignant cells and most commonly CD3 on T cells leading to an endogenous, HLA-independent, immune response against malignant cells. However, the use of bispecific antibodies in AML has been limited by the absence of highly specific leukemia-associated antigens leading to on-target, off-leukemia side effects as well as reduced efficacy due to antigen escape. Herein, we discuss the history and evolution of bispecific T cell engagers as well as various adaptations such as dual affinity retargeting antibodies, bi- and tri-specific killer engager antibodies. Common side effects including cytokine release syndrome and management thereof are highlighted. Lastly, we expound on the future direction and integration of such antibody-based therapies with other immunotherapies (programmed cell death-1 inhibitors and chimeric antigen receptor T cells).

## 1. Introduction

Acute myeloid leukemia (AML) is a clonal proliferation of myeloid hematopoietic stem cells (HSCs) defined by the presence of more than 20% blasts in the bone marrow [1]. For decades, the mainstay of treatment has included intensive multi-phased chemotherapy regimens for medically fit patients consisting of anthracycline/cytarabine-based induction chemotherapy followed by cytarabine-based consolidation, and potentially allogeneic hematopoietic cell transplantation (allo-HCT) [2]. Lower-intensity alternatives, including various targeted agents (e.g., the BCL-2 inhibitor venetoclax, the FLT3-inhibitor gilteritinib or IDH1/2 inhibitors) and hypomethylating agents (HMA), are often used for patients with significant medical comorbidities or elderly individuals who would not tolerate intensive induction chemotherapy [3,4]. The prognosis of an individual AML patient is dependent on multiple variables including a patient’s age and functional status as well as the type of AML and genetic factors [5]. Due to the poor prognosis of AML with a 5-year survival of only 28.7% [6], and difficulty of achieving a durable complete remission without allo-HCT, additional treatment options are warranted. Both molecularly targeted treatments and immunotherapies can be administered in parallel with chemotherapy or used alone for patients with relapsed or refractory (R/R) disease [7,8,9,10].

Antibody-based therapies target specific leukemic cell antigens. Bispecific T cell engagers (BiTEs) were one of the first antibody-based leukemia therapies engineered, with the anti-CD3 × CD19 antibody blinatumomab being the first agent in this class to have garnered FDA approval for the treatment of acute lymphoblastic leukemia (ALL) [11,12]. In this review, we will highlight antibody-based therapies, including bispecific antibodies, by discussing the history, mechanism of action, evolution of the antibody construct, the relevant clinical and preclinical literature, and side effects. Additionally, alternative constructs of bispecific antibodies, including dual affinity retargeting antibodies (DARTs), and bispecific killer cell engagers (BiKEs) will be discussed. A detailed review of the immunologic background would be beyond the scope of this review and we would like to refer the reader to previously published excellent reviews on the immunology and construct design [13].

## 2. Historical Overview and Safety Considerations

**Figure 1:**
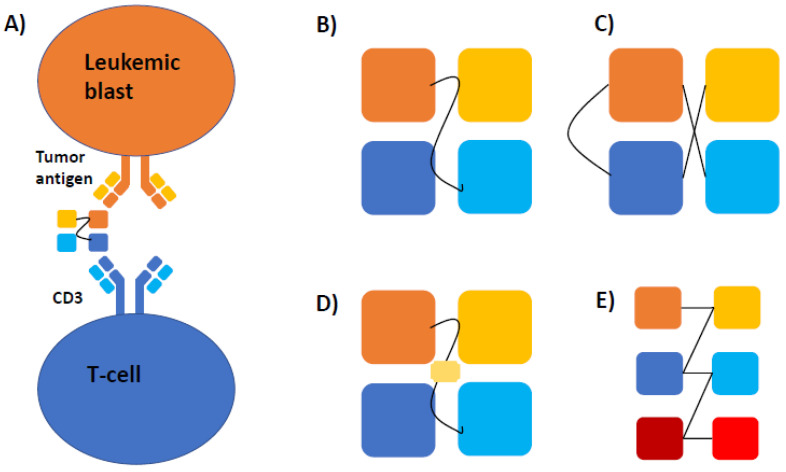
(**A**) Mechanism of action and basic construct of various bispecific antibodies. Bispecific antibodies consist of a single heavy and light chain of the variable region of a tumor-associated antigen (e.g., CD33 or CD123; shown as yellow and orange squares) and CD3 (illustrated as light and dark blue squares) leading to the formation of a cytolytic synapse between T-cells and leukemic blasts. (**B**) In their basic construct, these bispecific T-engaging antibodies (BiTEs) are connected by a linker molecule, which defines the flexibility of the construct and antigen-binding kinetics in conjunction with the specific antigens used. (**C**) Dual-affinity re-targeting molecules (DARTs) have a similar basic structure but include a disulfide linker for additional stability. (**D**) Bispecific (BiKEs) and (**E**) trispecific killer cell engagers (TriKEs) consist of either two (BiKE) or three (TriKE) variable antigen regions and activate natural killer cells either by binding to IL16 or containing an IL15 linker (yellow rectangle in (**D**)).

Bispecific antibodies are unique molecular constructs that allow for simultaneous binding of two antigens: a surface target on malignant cells and CD3 on T cells. While several modifications have been developed, the basic construct of various forms of bispecific antibodies has remained the same and is illustrated in Figure 1. Once both binding sites are engaged, T cells are activated and tumor cell lysis and cytokine release are triggered [14]. In 1985, there were two publications providing proof of concept that a hybrid antibody was able to activate effector T cells against a target antigen. The invariant region glycoprotein T3 on T cells was identified to serve as the T cell engager component of the bispecific antibody [15,16]. Bispecific antibody-directed T cell activation occurs independently of the major histocompatibility complex (MHC) and costimulatory pathway, allowing for cytotoxicity to occur despite MHC downregulation on malignant cells [17]. Blinatumomab was the first bispecific antibody approved for clinical use in R/R-B-cell ALL. It is a bispecific monoclonal antibody directed against CD3 on T cells and CD19 on blast cells. CD3 interacts with T cell receptors, facilitating signal transduction and activation of T cells to form an immune response [18]. CD19 is a cell surface marker on B cells that functions as a transmembrane protein and is used by blinatumomab to direct T cells against ALL blasts [19]. The randomized controlled trial compared blinatumomab with chemotherapy. Blinatumomab demonstrated improved overall survival (OS; 7.7 vs. 4.0 months; hazard ratio (HR) for death 0.71; 95% confidence interval (CI), 0.55 to 0.93; *p* = 0.01), as well as a longer median duration of remission, albeit with ≥grade 3 adverse events occurring in 86% in the blinatumomab group and 92% in the chemotherapy group [12]. Additionally, blinatumomab was proven to be efficacious in a single-arm study enrolling B-ALL patients in first or second complete remission (CR) with measurable residual disease (MRD) with a median OS of 36.5 months and an MRD-negative CR rate of 78% [11]. These studies provided the scientific rationale to further explore bispecific antibodies in other hematologic malignancies, including AML, by targeting antigens such as CD33, CD123, and CLEC12A [20]. Several early trials using antibodies to target those antigens in AML had limited efficacy and were terminated early [21,22]. However, recent advances in engineering technologies have led to a new generation of bispecific antibodies with promising early results, as discussed herein.

## 3. Mechanism and Structure

Bispecific antibodies are small molecules engineered to engage two antigens, the CD3 of T cells and a tumor associated antigen (TAA). Two single chain variable fragments (scFVs) generate the antigen specificity (Figure 1). scFVs are composed of a heavy and light chain that are connected via a linker sequence. The length of the sequence has a direct correlation with the flexibility of the antibody, binding both antigens simultaneously. Once bound to CD3, the T cell receptor is stimulated and directs a cytotoxic response to the bound tumor cell [23]. This highly specific process reduces off-target cytotoxicity, as the T cells will only be activated in the presence of their target cells [24]. One of the hallmark features of tumor cells is the evasion of host immune cell responses by the downregulation of cell surface antigens [25]. A particular challenge related to utilizing host adaptive immunity is the downregulation of MHC and costimulatory molecules on malignant cells. However, bispecific antibodies have demonstrated the ability to mount a potent effector response to tumor cells in an MHC-1- and costimulatory signal-independent manner [26]. Bispecific antibodies are capable of forming a cytolytic synapse between CD8 T cells regardless of MHC-1 expression on tumor cells [17].

The development of next-generation bispecific antibodies has focused on prolonging the half-life, as the small molecules are rapidly cleared from circulation by the kidney [27]. Innovations with blinatumomab yielded a construct including CD19 and CD3 scFVs fused to Fc antibody domain, termed CD19 half-life-extending (HLE) bispecific antibodies. In contrast to current bispecific antibodies that require continuous infusions, the HLE design could be suitable for once weekly dosing, as demonstrated both in vitro and in vivo [28]. Additionally, AMG 673, a novel HLE bispecific antibody that binds CD33 and CD3 with fused single chain IgG Fc, was studied in R/R AML by infusing two doses over 14 days, demonstrating longer half-life than prior bispecific antibody constructs [29]. One theorized concern with HLE formulations relates to adverse events (AEs), including cytopenias and cytokine-release syndrome (CRS). In the continuous infusion form, the bispecific antibodies are quickly cleared when infusions are stopped; conversely, HLE BiTEs would not be cleared as rapidly due to longer half-lives of up to 210 h [28,29]. There have been no head-to-head comparisons of the formulations that demonstrate variations in the rates of AEs.

After a better understanding of the mechanism of bispecific antibodies was gained by targeting leukemic antigens through utilizing the immune system for an anti-cancer effect, other variant constructs were developed to diversify the landscape of targeted therapies. Variant constructs to the bispecific engager include dual affinity retargeting antibodies (DARTs), as well as bi- and tri-specific killer engager antibodies (BiKEs and TriKEs). Their basic construct is shown in Table 1. DARTs use a diabody back bone with the addition of a c-terminal disulfide bridge that improves stabilization (Figure 1C). When compared head-to-head in vitro with CD19 antigen specificity, DARTs yielded a stronger B cell lysis and T cell activation [30]. BiKEs (Figure 1D) and TriKEs (Figure 1E) utilize the innate immune system by harnessing natural killer (NK) cells via CD16. CD16 is a receptor on NK cells that interacts with immunoglobulins, and upon activation will stimulate the production of cytokines, i.e., IL2, by NK cells [31]. Upon stimulation, the NK cells produce cytokines and invoke a cytolytic response against target tumor cells [32]. NK cells are inhibited when they interact with MHC-1. AML cells can express MHC-1, thus it was theorized that MHC-1 expression by AML cells would prevent a cytolytic response when exposed to TriKEs or BiKEs [33]. The first BiKE in AML targeted CD16 and CD33, and was able to induce NK cell activation against tumor cells regardless of MHC-1 presence and to eliminate AML cells [33]. In patients with myelodysplastic syndrome (MDS), host NK cells were activated in the presence of CD16 × CD33 BiKEs to target CD33+ MDS cells [34].

TriKEs have three components. Similar to BiKEs, there are two scFVs: CD16 engages NK cell and CD33 binds tumor cells. The third component is the addition of an IL-15 crosslinker to expand the NK response [35]. TriKEs with CLEC12A, which is expressed on AML and leukemic stem cells (LSCs), were able to reduce tumor burden in vitro and in mouse models, while successfully sparing HSCs [36]. In vitro, TriKEs enhanced NK cell cytotoxicity, degranulation, and cytokine release [37].

## 4. Antigen Targets

There are various antigens that could serve as targets for bispecific antibodies, but certain characteristics should be present for an antigen to be clinically relevant, such as high expression on malignant cells and high affinity binding [38]. Specific antigens in AML have been proposed and explored for their clinical utility. Table 2 provides an overview of preliminary results of selected clinical trials in AML.

### 4.1. CD33

CD33 is a myeloid differentiation antigen expressed on up to 90% of leukemic blasts and has the advantage of very limited, if any, expression outside of the hematopoietic system [40,41]. CD33 is the target for the antibody-drug conjugate gemtuzumab ozogamicin, which has garnered FDA approval in newly diagnosed CD33-positive AML [42]. CD33 was integrated as one of the scFV antigens in the bispecific antibody AMG330. AMG330 was assessed in vitro with CD33+ stem cells, and demonstrated a potent T cell recruitment, expansion, and cytotoxicity of leukemic cells [43]. Preclinical studies with CD33 expressing cells found that AMG330 cytotoxicity was not impacted by single nucleotide polymorphisms (SNPs) in CD33, nor adenosine triphosphate–binding cassette (ABC) transporter proteins [44]. Further exploring the effector:target (E:T) cell ratio, AMG330 was found to produce a strong cytotoxic response even at low levels of CD33 expression. There was an E:T correlation at high doses of AMG330 and high CD33 expression. When studied in vitro across the different stages of AML, AMG330 yielded the strongest effect in newly diagnosed AML with favorable risk versus R/R [45]. Despite this being an MHC-1- and costimulatory-independent process, AMG330 activity can be modulated by cell surface ligands. Expression of PD-L1 and PD-L2 by tumor cells decreased activation of T cells in the presence of AMG330. Conversely, when CD80 and CD86 were expressed, and CD28 co-stimulation occurred, T cell activation was enhanced [46]. Demonstrated in vitro, AML cells upregulated the expression of checkpoint inhibitor receptors, such as PD-L1, in the presence of AMG330 secondary to proinflammatory cytokine release. Through blockade of PD-1/PD-L1 interaction, AMG330 enhanced cell lysis and T cell proliferation, pointing towards the clinical utility of checkpoint inhibitors as potential co-administered immunotherapies [47]. AMG330 was administered in phase 1 trials with R/R AML, which expresses CD33 in 99% of cases, and established AMG330′s anti-leukemic activity in heavily pretreated patients [48,49]. Preliminary results from an ongoing, open-label, phase I trial that enrolled 55 patients with R/R-AML (NCT02520427) were recently presented. Eight of the 42 (19%) evaluable patients demonstrated a response (three CR, four CRs with incomplete hematologic recovery (CRi), and one morphologic leukemia free state). Notably, half of the responders had already undergone more than four lines of prior therapy [49]. The safety profile of AMG330 appeared manageable, with CRS (67%; 13% with grade ≥ 3) and nausea (20%) being the most frequent AEs.

AMV564, another CD3 × CD33-targeting bispecific antibody, was studied in murine models of AML. Not only did it produce a potent anti-tumor effect in both the bone marrow and peripheral blood, but it also prolonged survival [50]. In phase 1 clinical trials, AMV564 had significant anti-leukemic activity via T cell activation [51]. When evaluated in R/R AML, AMV564 was able to achieve anti-leukemic activity and has the advantage of a prolonged half-life. AMV564 activates T cells in an antigen-specific manner, which minimizes off target effects and eliminates myeloid blasts regardless of antigen expression level or disease stage [52]. Myeloid-derived suppressor cells (MDSC) act to inhibit T cell response. However, AMV564 is able to deplete MDSC via the binding of CD33, thus promoting T cell activation in AML and MDS. In patients, there was a dose-dependent relationship of depleting MDSC and activating T effector cells [53].

AMG673, a CD3 × CD33 HLE bispecific antibody, has also entered clinical trials in R/R AML. As this is an HLE construct, patients underwent two infusions on days 1 and 5 of each 14-day cycle. AMG673 demonstrated a reduction in blast burden in 11 out of 27 patients (41%) with a single patient (4%) achieving a CR. However, half of the patients had varying grades of CRS [29]. JNJ-67571244, a CD3 × CD33 bispecific antibody, was tested in in vitro, murine, and primate models and binds the C2 domain of CD33 to induce a T cell response independent of SNPs. There was a significant anti-tumor response leading to the depletion of CD33+ blast cells. With promising data, it has entered phase 1 clinical trials in R/R AML and high-risk MDS [54]. Additional clinical trials are underway with the CD33 targeting bispecific antibody GEM333. Table 3 provides an overview of active clinical trials of bispecific antibodies in AML.

BiKEs targeting CD33 and CD16, the Fc receptor on NK cells, have also been developed [33]. An important downregulator of CD16 is ADAM17, a transmembrane CD16 sheddase [33]. Thus, when studied in vitro, the CD16 × CD33 BiKE was combined with an ADAM17 inhibitor. The CD16 × CD33 BiKE activated NK cells against de novo and refractory AML cells, and when combined with an ADAM17 inhibitor was able to overcome the MHC-1 inhibitory signals, when MHC-1 was expressed in AML cells [33]. In a different study of MDS samples, the CD16 × CD33 BiKE demonstrated two key findings. First, there was a reversal of the MDSC-induced suppression of NK cells. Secondly, the NK cells mounted a targeted lysis of CD33+ MDS cells [34]. A TriKE was constructed with a IL15 crosslinker combined with the scFvs against CD16 × CD33, known as 161533. IL15 aids in the activation of NK cells [55]. Compared to a BiKE with the same scFvs (BiKE 1633), the TriKE model produced a stronger NK response with respect to cytotoxicity, degranulation, and cytokine production, a presumed result secondary to IL-15 promoting NK cell development and survival [37].

### 4.2. CD123

CD123, an IL-3 receptor alpha chain, is expressed at high levels on leukemic blasts and relatively deplete on normal HSCs. Despite being an IL-3 receptor, when activated on leukemic cells, it does not mediate the normal IL-3 signal transduction [56]. XmAb14045, a bispecific antibody with antigen specificity for CD3 and CD123, was first developed and studied in animal models, yielding a T cell-mediated killing of the AML cell lines KG-1a and TF-1, both in the peripheral blood and bone marrow. With the addition of an Fc domain, XmAb14045 had a significantly longer half-life at 6.2 days [57]. Preliminary results from a multicenter, open-label phase 1 dose-escalation study of XmAb14045-01 (vibecotamab) in 106 patients with relapsed or refractory hematologic malignancies (104 patients with R/R AML) showed an overall response rate (ORR) of 14% (7 out of 51 patients) at the higher dose level, with two patients achieving a CR [58]. However, no patients treated with the lower dose experienced an objective response. Whether the stable disease seen in 71% of patients translates into any clinically meaningful benefit remains to be evaluated during extended follow-up. Notably, 58.5% of patients developed CRS with 15% of CRS events being ≥ grade 3. Correlative studies also suggested that patients with lower disease burden and specific T-cell subtypes had a higher likelihood of response, while CD123 expression on AML blasts was not associated with response [58].

JNJ-63709178, another bispecific antibody with an Fc domain targeting CD123, is currently undergoing phase 1 clinical trials (NCT02715011) [59]. APVO436, yet another anti-CD123 bispecific antibody, is in clinical trials of AML and MDS (NCT03647800). Interestingly, this particular construct has the potential for less CRS when compared to the DART MGD006 (discussed below), secondary to lower induction of T cell cytokine release, yet APVO436 was still able to induce T cell proliferation and development of memory T cells with cytolytic function [60]. However, preliminary results from an ongoing phase I study (NCT03647800) showed limited efficacy with only 2 out of 19 patients experiencing a blast reduction [61]. SAR440234 is in clinical trials and modeled similarly to JNJ-63709178 with an Fc domain but clinical data is still under review at this time.

MGD006 is an anti-CD123 DART that prompts T cell activation via CD3 engagement and elimination of CD123 positive AML cells. Preclinical studies engineered MGD006 to have a higher affinity to CD123 than CD3, for preferential binding of leukemic cells. Circulating CD123 positive blasts were cleared at low doses of MGD006. Bone marrow cellularity was also preserved with drug administration, and maintenance of normal HSCs [62]. MGD006 was demonstrated in another preclinical trial to produce not only cytotoxicity, but also T cell receptor diversification [63]. Clinical trials with MGD006 are evaluating a dose escalation regimen in R/R AML as well as MDS [64].

Flotetuzumab, a CD123 × CD3 bispecific DART, has also entered clinical trials for R/R AML and demonstrated antileukemic responses in a subset of heavily pretreated patients [65]. The results from a subgroup of 30 AML patients with primary induction failure or early relapse enrolled in a phase 1/2 trial (NCT02152956) demonstrated an ORR (defined as CR/CR with hematologic recovery/CRi) of 30% with a median overall survival of 10.2 months among responders and a safety profile comparable to other bispecific antibodies [65,66]. Highlighting the importance of translational studies, the authors were able to identify transcriptomic signatures in the bone marrow that predicted responses to flotetuzumab. Notably, studies have demonstrated the upregulation of PD-1/PD-L1 expression on T cells following activation by flotetuzumab. Flotetuzumab, combined with MGA012, an anti-PD-1 antibody, is currently being studied in clinical trials for R/R AML and hypothesized to exhibit enhanced T cells activation compared with flotetuzumab alone [67].

Talacotuzumab, a monoclonal antibody against CD123, has been studied in a phase 2/3 trial in combination with the HMA decitabine vs. decitabine alone. Among 316 older AML patients ineligible for intensive chemotherapy treated at target dose, the combination therapy yielded median OS and CR rates that were similar to decitabine alone (median OS: 5.36 (95% CI: 4.27–7.95) months with combination vs. 7.26 (6.47–8.64) months for decitabine alone (hazard ratio: 1.04; 95% CI: 0.79–1.37; *p* = 0.78); CR: 15% vs. 11%; odds ratio: 1.4; 95% CI: 0.6–3.6; *p* = 0.44) [68]. Similarly, a smaller trial of talacotuzumab monotherapy in HMA-refractory AML and MDS patients showed that blockade of CD123 in this population was less effective, which is presumed to be the result of altered NK and T cell function as disease progresses. Another limitation were AEs, including infection and cytopenias [22]. As talacotuzumab is a monoclonal and not a bispecific antibody, it is unclear how results can be translated across different types of immunotherapies. However, it does highlight the challenges regarding target antigen selection in myeloid malignancies.

### 4.3. CLL-1/CLEC12A

CLEC12A, also known as C-type lectin-like molecule-1 (CLL-1), is a myeloid differentiation antigen expressed by 90–95% of leukemic blasts [69,70]. Additional support for using CLEC12A as an immunotherapy target comes from a recent expression study in pediatric AML patients that showed that the combination of CD33 and CLEC12A was the most frequently upregulated one in pediatric AML samples, making it a potential therapeutic target with limited on-target off-leukemia side effects [71]. CLEC12A has been evaluated as an immunotherapy target both with antibody-drug conjugates (ADCs) and bispecific antibodies. The first bispecific antibody engineered to target CLEC12A demonstrated a dose-dependent activation of T cells and decrease in the CLEC12A positive cell population [72]. Unlike CD33 and CD123, CLL-1 expression is limited to HSCs, theoretically providing enhanced hematopoietic recovery after therapy [73]. MCLA-117, a bispecific antibody with antigen specificity to CLEC12A × CD3, was studied in preclinical models and demonstrated efficient blast lysis even at low E:T ratios [70]. An additional in vitro study showed a potent activation and redirection of T cells against CLEC12A AML cells, even at low E:T ratios on cells with low CLEC12A expression. The infusion schedule also supported a weekly or biweekly interval as the structure backbone has a full-length IgG1 extending its half-life [74]. MCLA-117 is currently under investigation in clinical trials (NCT03038230).

As LSCs may lack CD33 expression in relapsed AML, a TriKE targeting CLEC12A was developed, as up to 70% of CD33-negative AML cells will express CLEC12A at relapse. Compared to the CD33 target, the CLEC12A TriKE had fewer off target effects on peripheral blood mononuclear cells (PBMC) with similar efficacy in NK cell activation [35]. This was further explored in mouse models, demonstrating that the CLEC12A TriKE induced a robust NK cell response, killing AML cells while sparing normal HSCs [36]. The DART known as CLEC12A-ENG.CD123IL7Ra was synthesized to target CLEC12A with the addition of IL7Ra to the CD123 portion of the DART. In in vitro and murine models, it showed an increased target cells recognition as well as enhanced survival and activation of T cells [75].

### 4.4. FLT3

The *FLT3* mutation status of newly diagnosed AML has become essential in the management and prognosis of AML [2]. FLT3 is a receptor tyrosine kinase that is targeted by small molecule inhibitors such as gilteritinib and midostaurin [76,77]. An anti-FLT3 × CD3 bispecific antibody was constructed to exploit the same receptor expressed on AML cells. Interestingly, the bispecific antibody when compared to monospecific anti-FLT3 antibodies was superior with diminishing PBMC [78]. AMG427, an anti-FLT3 × CD3 bispecific antibody, demonstrated T cell-dependent cellular cytotoxicity. When cells were exposed to AMG427, there was a dose-dependent upregulation of PD-1 expression on T cells. By combining PD-1 blockade with AMG427, the drug’s potency was restored, allowing for enhanced targeting and clearance of leukemic cells [79]. Most recently, bispecific antibody 7370 was analyzed for its unique qualities in vitro and in vivo, as it is able to target FLT3 regardless of mutational status at low E:T ratios [80].

## 5. Cytokine Release Syndrome and Other Toxicities

CRS remains a major adverse event with bispecific antibodies and was first described with the use of blinatumomab [12]. CRS is a unique toxicity that is frequently observed in immunotherapies that lead to a nonspecific activation of the immune system with release of cytokines such as IL-6, INFγ, and TNFα into circulation, prompting further activation of lymphoid and myeloid cells. This mass activation and cytokine release manifests as potentially life-threatening end organ damage in multiple systems, with presentations including constitutional symptoms, rash, nausea/vomiting, diarrhea, hypoxemia, tachycardia, hypotension, azotemia, hepatic injury, and altered mental status [81]. Therefore, some clinical trials have adopted risk mitigation measures to avoid or minimize CRS. For example, when CRS was identified in a trial with AMG330, symptoms would typically resolve within one day of stopping AMG 330 and treatment with steroids, fluids and vasopressor support [48].

The management of CRS is based on a grading system from grade 1 (nonspecific, non-life-threatening) to grade 5 (death). Grade 1 and 2 CRS are most commonly managed with general supportive measures. Grade 3 or greater often require further interventions including steroids, or the anti-IL-6 antibody tocilizumab. Tocilizumab is limited to peripheral CRS treatment, as it does not penetrate the blood–brain barrier, thus neurologic manifestations of CRS are typically managed with high doses of dexamethasone [81]. Reassuringly, in vitro data showed that while dexamethasone reduces circulating cytokine levels following blinatumomab treatment, this did not negatively affect blinatumomab’s therapeutic cytotoxicity [82]. As CRS is an expected adverse event with bispecific T-cell engagers, recommendations to mitigate the incidence and severity of CRS have been proposed and include a step-wise dose increase as well as prophylactic treatment with dexamethasone [40].

Another described toxicity of bispecific antibodies is immune-effector cell-associated neurological syndrome (ICANS). Though more likely to occur with chimeric antigen receptor (CAR) T cells, it has also been described with blinatumomab at a rate of 9% [12,83]. Thought to be the result of excessive cytokine release and blood–brain barrier disruption, ICANS presents with mental status changes, such as inattention and obtundation, or higher cognitive manifestations including agraphia and aphasia [39]. Notably, this can occur in tandem with CRS or independently [84]. Blinatumomab has also been associated with other neurologic adverse events including encephalopathy, tremor, dizziness, and confusion [85]. Treatment usually includes steroids, and if there is coinciding CRS, tocilizumab (limited to peripheral action) and other immunosuppressing agents are considered [86]. Although data are limited, ICANS and neurologic side effects appear to be less common in AML patients treated with bispecific antibodies [29,66].

## 6. Overcoming Limitations of Bispecific Antibodies and Future Directions

Short half-lives necessitating the administration of bispecific antibodies as a continuous infusion leads to a significant burden on patients and the healthcare system. The addition of a Fc domain in HLE constructs helps to prolong their half-life. Bispecific tetravalent antibodies can increase the molecular weight to enhance the half-life by avoiding rapid renal clearance, which is typically observed with current bispecific antibodies [87]. BC133, a tetravalent bispecific antibody, demonstrated a >10-fold potency when compared with heterodimer counterparts, without inducing a targeted response against normal HSCs [88].

A major mechanism of resistance to bispecific antibodies is immune escape by upregulation of PD-1 on activated T-cells. Upregulation of PD-L1 has been documented in AML cells treated with AMG330 and can be overcome by PD-1/PD-L1 blockade, which has led to a phase 1 trial combining the anti-PD-1 antibody pembrolizumab with AMG330 (NCT04478695), as well as the combination of flotetuzumab with the novel anti-PD-1 antibody MGA012 [47,67]. The role of MDSCs in the tumor microenvironment in AML is evolving, but studies with anti-CD16 × CD33 BiKEs and AMV564 have identified MDSCs as additional targets, whose elimination restores physiologic immune function and contributes to tumor cell elimination [34,89]. Bifunctional checkpoint inhibitory T cell–engaging (CiTE) molecules are antibody constructs that combine the backbone of bispecific antibodies with the addition of a checkpoint inhibitor to block upregulated inhibitor molecules on effector T cells, allowing for enhanced engagement and potent targeting of leukemic cells [90]. Correlative studies from the flotetuzumab trial also showed a highly immunosuppressive tumor microenvironment that is characterized by upregulation of inhibitory immune checkpoint genes such as *ICOS* and *NOTCH2*, which can be stimulated by flotetuzumab [66]. Additionally, higher levels of PD-L1 expression have been shown to be more resistant to treatment with flotetuzumab, providing additional support for trials combining PD-1/PD-L1-targeted therapies with flotetuzumab or other bispecific antibodies [66,91]. In AML or MDS patients who have *TP53* mutations, PD-L1 expression is increased and can facilitate immune evasion via an immune-privileged phenotype [92]. Evaluation of *TP53* mutational status in addition to a gene panel-based immune signature and conventional cytogenetic risk stratification in patients undergoing therapy with flotetuzumab could become a step towards a more individualized treatment approach and increase the likelihood of response [66]. However, whether similar predictive biomarkers can be identified for other bispecific antibodies remains to be seen.

Other innovations have focused on combining therapies and adaptations to current constructs. CAR T cell therapies are in development for myeloid malignancies, but their use has been limited by on-target off-leukemia effects. Integrating bispecific and CAR T cell technologies has become a recent focus. BissCAR T cells are CAR T cells that have a bispecific component. Recently, a CAR T cell with bispecific specificity to CD13 and TIM3 demonstrated the ability to target cells with both CD13 and TIM3 expression but spared those with either antigen alone. The rationale for using TIM3 as a target is its higher expression on exhausted T cells and LSCs compared to normal HSCs, which also reduced the cytotoxicity of CAR T cells in vivo [93,94]. Nonetheless, it continued to have a more targeted antileukemic effect when combined with bispecific engagers [95]. Another multimodal therapeutic approach includes combining venetoclax with CD123 bispecific T cells, though in vitro models demonstrated potential adverse effects such that both AML and healthy T cells had decreased survival [96]. The future of immunotherapy and bispecific antibody technologies will depend on continued in vitro studies analyzing how different immunotherapies can be combined in order to optimize anti-leukemic effects, while minimizing the off-target toxicity of these therapies. It will also be interesting to see if advances in biotechnology such as gene-editing with CRISPR/Cas-9 can lead to even more specific and potent agents [97].

A major challenge remains the determination of the optimal timing for treatment with bispecific antibodies. Preliminary data suggest that patients with lower tumor burden are more likely to achieve a response than patients with higher numbers of circulating blasts, which could also reduce the incidence of severe CRS [58]. Persistent MRD has been shown to be associated with higher rates of relapse and adverse outcomes following intensive chemotherapy and allo-HCT [98,99,100]. Based on data from ALL patients treated with blinatumomab showing that it is highly effective in the eradication of MRD, bispecific antibodies in AML might be especially useful in the setting of MRD-positive disease following intensive chemotherapy or allo-HCT as well [11]. As AML relapse has been associated with the persistence of LSCs, which are characterized by the expression of CD123, using bispecific antibodies against CD123 such as flotetuzumab could be an effective way to induce deeper remissions and prolong time to or even eliminate relapse [66,93,101]. Interestingly, none of the patients with late relapse achieved a response with flotetuzumab, which could be associated with a distinct immunologic milieu between patients with higher expression rates of inflammatory cytokines, PD-L1 and CD123 in patients with primary induction failure and early relapse compared to patients with late relapse [66]. As patients with adverse- and intermediate-risk cytogenetics seemed to respond better to flotetuzumab, this agent might have a unique role in this very high-risk population of patients, but additional studies are needed.

A serious safety concern related to using antigens such as CD123 that are expressed on normal hematopoietic cells remains prolonged neutropenia [102]. While adverse events related to lymphopenia can be mitigated by the administration of immunoglobulins, prolonged neutropenia can pose a substantial risk of mortality. However, rates of grade ≥ 3 neutropenia with flotetuzumab were low, at 3.4%. Additionally, the fact that 57% of patients with a CR were experiencing count recovery while receiving flotetuzumab suggests that it does not lead to a prolonged or significant effect on normal hematopoiesis [66]. Whether this also applies to other constructs remains to be evaluated in clinical trials. Preliminary data showed rates of grade ≥ 3 febrile neutropenia of 7% and 25% with AMG 673 and APVO436, respectively [29,61]. While this is higher than with blinatumomab, it appears manageable, but longer follow up and data on other constructs are necessary [12].

CRS remains a major concern and novel constructs have focused on alternative targets such as TIM3 or mitigating CRS by co-treatment with immunomodulatory agents such as the Janus kinase inhibitor ruxolitinib or the TNFα inhibitor etanercept [40,95,103,104]. However, additional preclinical and clinical studies are needed to assess whether these agents are both effective in preventing CRS and do not impair therapeutic efficacy. Additionally, biomarkers predictive of the onset and severity of CRS are needed to potentially guide timing of tocilizumab or steroid administration. In the flotetuzumab trial, baseline CD4 count predicted CRS severity, while blast count or CD123 expression was not associated with CRS severity [66].

Finally, resistance to bispecific antibodies occurs via immune escape by down-regulation of the target antigen or via the up-regulation of inhibitory immune checkpoints to create a permissive, immunosuppressed microenvironment. While downregulation of target antigens has been documented in CD19-directed CAR T-cells and with blinatumomab, this has not been observed with bispecific antibodies in AML thus far, but additional studies with longer follow up are needed [40,105,106]. Targeting multiple different tumor antigens simultaneously or at the time of progression might be a possibility to address this resistance mechanism [83]. However, no data supporting the sequential use of various different bispecific antibodies in AML have been published to date.

## 7. Conclusions

Following initially disappointing results, several bispecific antibody constructs have been developed and tested in early phase clinical trials with encouraging results. CD33 and CD123 appear to be the most promising targets, although their use may be limited by side effects, antigen escape, and potential nonselective activation of the immune system. Several challenges remain pertaining to the selection of patients based on genetic and immune markers and optimal timing of bispecific antibody use along the disease course (primary induction failure vs. MRD eradication vs. manifest relapse). CRS, neurotoxicity, and myelosuppression remain major challenges, and modifications to the construct itself or combination with TNF-α or JAK inhibitors are ongoing.

## Figures and Tables

**Table 1:** Overview of bispecific antibody and variant construct components.

Construct	Components
Bispecific Antibody	2 scFVs: CD3 of T cells and antigen target
DARTs	2 scFVs: CD3 of T cells and antigen target, with addition of disulfide bridge
BiKES	2 scFVs: CD16 of NK cells and antigen target
Trikes	2 scFVs: CD16 of NK cells and antigen target and IL-15 crosslinker

**Table 2:** Summary of previously published trials of bispecific antibodies in AML.

Author	Drug (Construct)	Patient Population	Outcomes
Uy [27]	Flotetuzumab (anti CD3 × CD123 DART)	92 R/R-AML patients	Primary induction failure or early relapse cohort (*n* = 30): Efficacy: 27% with CR/CRh; median OS 10.2 months among responders Safety: 100% CRS (3% ≥ grade)
Ravandi [28]	AMG 330 (anti-CD3 × CD33 BiTE)	55 patients with R/R-AML	Efficacy: 19% ORR (7% CR) Safety: 60% CRS
Subklewe [39]	AMG 673 (Half-Life Extended Anti-CD3 × CD33 BiTE)	30 patients with R/R-AML	Efficacy: 44% with bone marrow blast reduction Safety: 50% CRS (13% ≥ grade 3)
Ravandi [30]	Vibecotamab (XmAb14045; anti CD3 × CD123 BiTE)	104 R/R-AML, 1 B-cell ALL, and 1 CML	Efficacy: 14% ORR (4% CR); 71% SD Safety: 59% CRS (15% ≥ grade 3)
Watts [31]	APVO436 (anti CD3 × CD123 BiTE)	22 R/R-AML and 6 R/R-MDS	Efficacy: 2 patients with blast reduction Safety: edema (32%), febrile neutropenia (29%), infusion reaction (21%), CRS (18%)

**Table 3:** Overview of current trials and targets.

Drug	NCT	Patient Population	Target	Phase
AMG330	NCT02520427	Relapsed/Refractory AML, Minimal Residual Disease Positive AML, MDS	CD3 × CD33 bispecific antibody	1
AMV564	NCT03144245	Relapsed/Refractory AML	CD3 × CD33 bispecific antibody	1
AMV564	NCT03516591	MDS	CD3 × CD33 bispecific antibody	1
AMG673	NCT03224819	Relapsed/Refractory AML	CD3 × CD33 bispecific antibody	1
GEM333	NCT03516760	Relapsed/Refractory AML	CD3 × CD33 bispecific antibody	1
JNJ-67571244	NCT03915379	Relapsed/Refractory AML, MDS	CD3 × CD33 bispecific antibody	1
GTB-3550	NCT03214666	Relapsed/Refractory AML, MDS, Advanced Systemic Mastocytosis	CD16/IL-15/CD33 TriKE	1/2
APVO436	NCT03647800	Relapsed/Refractory AML, MDS	CD3 × CD123 bispecific antibody	1
XmAb14045	NCT02730312	CD123 Expressing hematologic malignancies	CD3 × CD123 bispecific antibody	1
JNJ-63709178	NCT02715011	Relapsed/Refractory AML	CD3 × CD123 bispecific antibody	1
SAR440234	NCT03594955	Relapsed/Refractory AML, MDS, B-ALL	CD3 × CD123 bispecific antibody	1/2
MGD006	NCT02152956	Relapsed/Refractory AML, MDS	CD3 × CD123 DART	1/2
MCLA-117	NCT03038230	Relapsed/Refractory AML and newly diagnosed elderly AML	CD3 × CLEC12A bispecific antibody	1
AMG427	NCT03541369	Relapsed/Refractory AML	CD3 × CD135(FLT3) bispecific antibody	1

## Data Availability

Not applicable.
